# Three-dimensional culture of HeLa-FUCCI cells for study of bystander cell-cycle effect of high LET particles

**DOI:** 10.1093/jrr/rrt166

**Published:** 2014-03

**Authors:** Yuka Sakamoto, Kiichi Kaminaga, Yukiko Kanari, Miho Noguchi, Akinari Yokoya

**Affiliations:** 1 Ibaraki University, Japan; 2 JAEA, Japan

**Keywords:** bystander effect, 3D cell culture, spheroid, FUCCI-HeLa, cell-cycle arrest

## Abstract

It has been recognized that bystander effect is one of the key factors for radiobiological effects, particularly in low-dose region. Although <1% of cell nuclei were actually traversed by an alpha particle, 30% of the cells showed an increased frequency of sister chromatid exchanges at very low dose (0.31 mGy) of alpha particles exposed to a CHO culture dish [ 1]. Since then, a number of studies including high LET microbeam experiments have revealed that the bystander effect is possibly mediated through both gap-junction signal transfer and releasing bystander-transmitter molecules from the irradiated cell into medium. Although it has particularly been given considerable attention to the latter process, however, the signal transfer through medium seems very specific to the artificially system of monolayer culture dishes, which are substantially different from *in vivo* system in which cells contact each other to form a functionally three-dimensional (3D) structure. Bystander signals must mainly be transferred through gap junctions.

In order to examine bystander effects in the 3D cell system, we have developed a HeLa-FUCCI spheroid system. FUCCI (Fluorescent Ubiquitination-based Cell Cycle Indicator) cells show specific colors of cell nuclei depending on cell cycle. Thus, we can easily trace cell-cycle modifications by irradiation. We observed bystander cell-cycle delay as preliminary tests using monolayer culture of the HeLa-FUCCI cells. It will be very interesting to examine whether the cell-cycle effect also appear in the 3D cell system exposed to single high LET particles. We have studied suitable conditions for the spheroid culture, such as size of spheroids and methods of stable fixing a spheroid in a dish to perform the microbeam irradiation, and observation of the cell cycles of each cell in a spheroid after irradiation using time-lapse micro-imaging technique. The first day of the culture, single cells were seeded in a commercial 96-well multi-plate.

A typical spheroid image observed for 3 days after seeding was shown in Fig. 1. The cells substantially formed a 3D structure of spheroid, in which the cells showed different cell cycle as shown by green (S/G2) and red (G1). This result indicates that the cells in a spheroid keep cell division to grow. We further investigate the effect of high LET particle irradiation on cell cycle in a spheroid in the future.

**Fig. 1.**
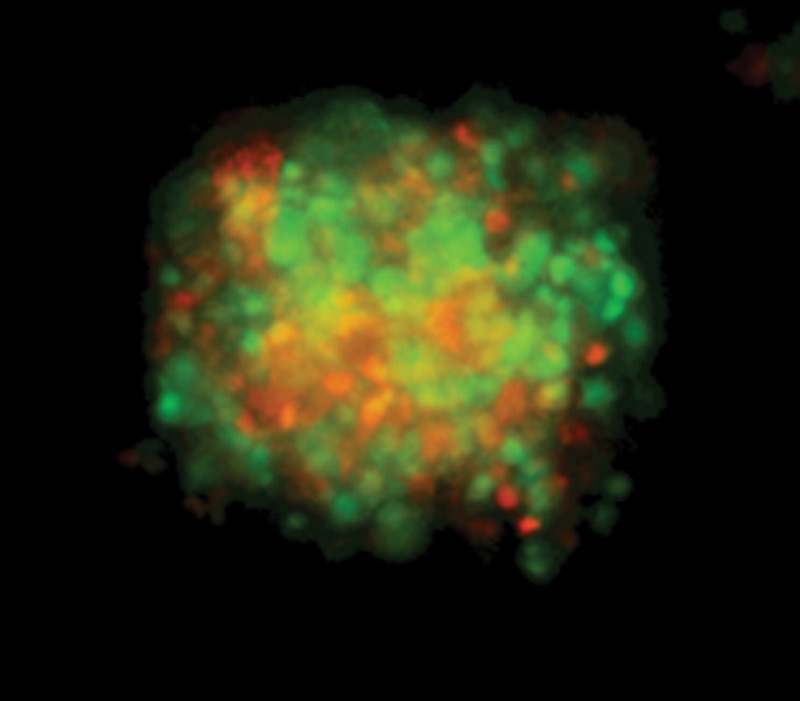
Spheroid after 3-day culture. Red and green cells are in G1 and S/G2 phase, respectively. The cells form a globular three-dimensional structure with about a few hundred micrometer.

Spheroid after 3-day culture. Red and green cells are in G1 and S/G2 phase, respectively. The cells form a globular three-dimensional structure with about a few hundred micrometer.
